# Supplementary material for: Histone H3 Lysine 4 and 27 Trimethylation Landscape of Human Alzheimer’s Disease
Source: Cells. 2022 Feb 19;11(4):734. doi: 10.3390/cells11040734 (PMC8870338; doi:10.3390/cells11040734)
Supplement: Supplementary file 1 [file cells-11-00734-s001.zip › cells-1566238 supplementary copy/cells-1566238 supplementary.pdf]

Supplementary materials

Table S1. Tissue donor information. Group, Age, sex, Braak stage, MMSE score and post-mortem delay (PMD) are reported for each of tissues donor used in the present study.

NPID	Age	Sex	Braak	Last MMSE	PMDelay
Control					
NP 12/161	69	male	1	Not available	48
NP13/081	70	male	1	Not available	48
RI110/04	86	male	1	Not available	96
RI 04/158	83	male	0	Not available	24
RI 03/211	85	male	0	Not available	24
NP 12/132	67	female	1	Not available	48
NP 13/074	70	female	1	Not available	24
RI 04/079	84	female	1	Not available	24
RI 03/205	84	female	1	Not available	48
Severe					
NP 14/115	75	male	5	0/30	46
NP 14/151	72	male	6	0/30	54
NP 14/109	78	male	6	0/30	34
NP 14/050	82	female	6	0/30	18
NP 14/033	77	female	5	0/30	31.25
NP128/14	61	female	6	Not available	23

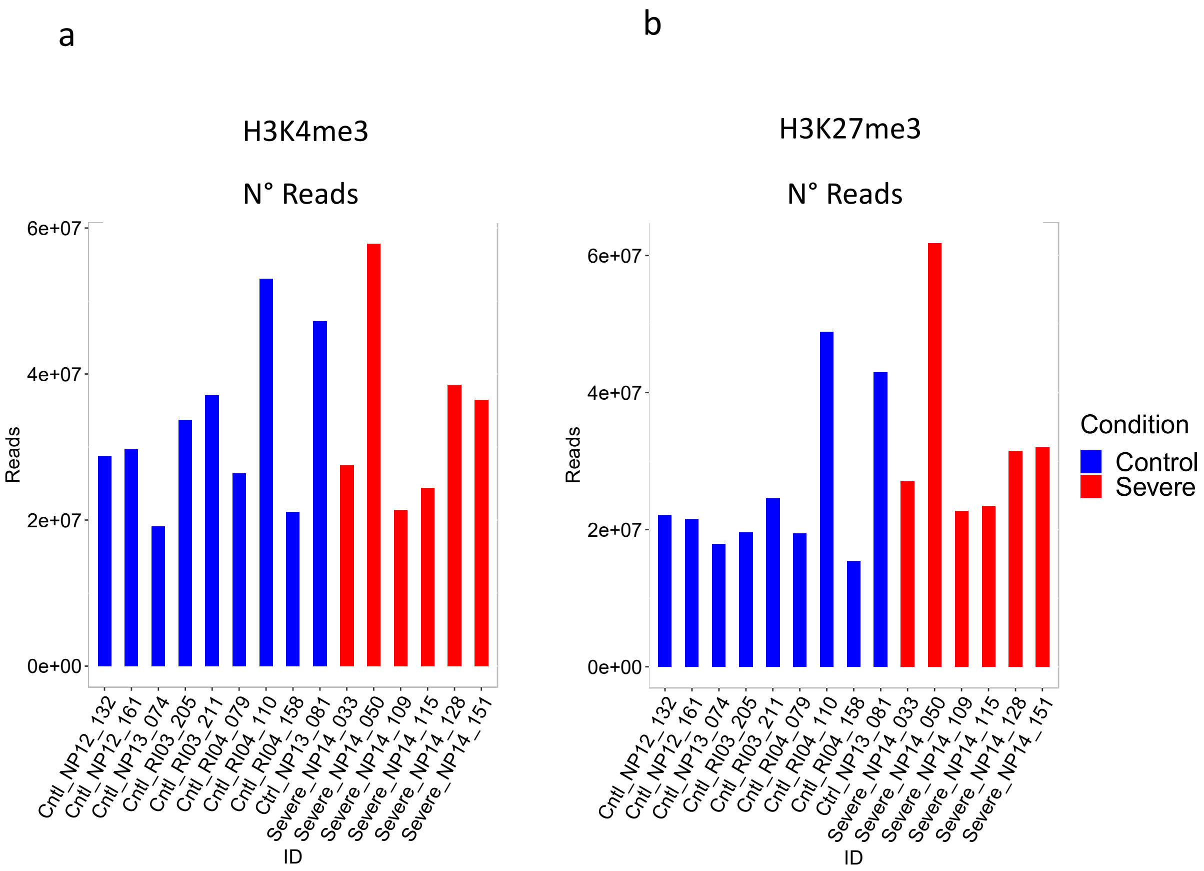

Figure S1. Number uniquely mapped and properly paired reads. Number of reads for H3K4me3 (a) and H3K27me3 (b). Control samples are in blue, severe AD in red.

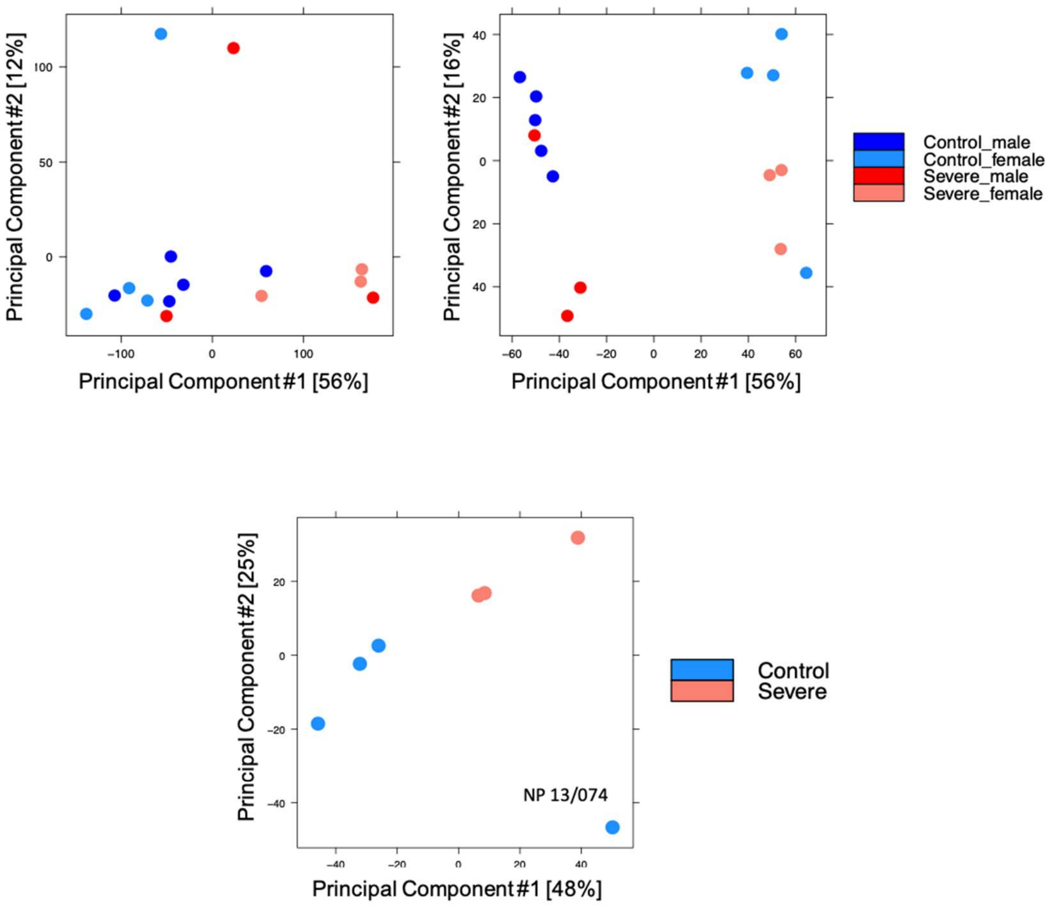

Figure S2: Principal Component Analysis.

(a) PCA using TMM normalized count of H3K4me3 dataset across 54,800 genomic regions.
(Control male in blue, Control female in light blue, Severe AD male in red, Severe AD female in pink)
(b) PCA using TMM normalized count of H3K27me3 dataset across 36,669 genomic regions.
(Control male in blue, Control female in light blue, Severe AD male in red, Severe AD female in pink)
(c) PCA relative to female H3K27me3 dataset. Control samples are in light blue, severe AD in pink.
